# Changes in the epidemiological patterns of respiratory syncytial virus and human metapneumovirus infection among pediatric patients and their correlation with severe cases: a long-term retrospective study

**DOI:** 10.3389/fcimb.2024.1435294

**Published:** 2024-09-02

**Authors:** Lu Kuang, Tiantian Xu, Changbing Wang, Jiahui Xie, Yingying Zhang, Min Guo, Zhuofu Liang, Bing Zhu

**Affiliations:** ^1^ Center Laboratory, Guangzhou Women and Children’s Medical Center, Guangzhou Medical University, Guangzhou, China; ^2^ Clinical Laboratory, Guangzhou Women and Children’s Medical Center, Guangzhou Medical University, Guangzhou, China

**Keywords:** respiratory syncytial virus, human metapneumovirus, acute respiratory tract illness, intensive care, pediatric

## Abstract

**Objectives:**

We aim to investigate the prevalence of respiratory syncytial virus (RSV) and human metapneumovirus (hMPV) among pediatric patients with acute respiratory tract illness (ARTI) in southern China both pre- and post-COVID-19 pandemic, as well as identify associated risk factors for severe infections.

**Methods:**

The study conducted a real-time PCR analysis on hospitalized children with ARTI from 2012 to 2023, specifically targeting RSV, hMPV, and other respiratory pathogens. Additionally, demographic data was collected during this analysis.

**Results:**

The prevalence of RSV occurs triennially, and likewise, the temporal pattern of hMPV outbreaks mirrors that of RSV. The peak infection rates of RSV and hMPV occurred during and following the implementation of COVID-19 epidemic prevention and control measures. The incidence of RSV infection exhibited bimodal peaks in 2022, while hMPV demonstrated seasonal peaks during the spring, fall, and winter periods post-COVID-19 pandemic. After the COVID-19 outbreak, there has been an upward trend in the proportion of female patients and patients aged one year and older presenting with ARTI, RSV infections, and hMPV infections. Infant (OR = 4.767, 95%CI: [3.888–5.846], *p* < 0.0001), presence of co-infection (OR = 0.540, 95%CI: [0.404–0.722], *p* < 0.0001), and existence of comorbidities (OR = 1.582, 95%CI: [1.285–1.949], *p* < 0.0001) was the risk ratio for the severity of RSV infection. Children infected with hMPV under the age of 1 year (OR = 0.322, 95%CI: [0.180 – 0.575], *p* < 0.0001), as well as those with comorbidities (OR = 8.809, 95%CI: [4.493 – 17.272], *p* < 0.0001), have a higher risk of developing severe illness.

**Conclusion:**

The changing epidemiological patterns have the potential to lead to widespread severe outbreaks among children, particularly those with underlying medical conditions who may experience more severe symptoms. Conducting surveillance for *pneumoviridae* viruses in children is an imperative measure to establish a robust foundation for future epidemic prevention and treatment strategies.

## Introduction

*Pneumoviridae* comprises large enveloped negative-sense RNA viruses, which was formerly a subfamily within the *Paramyxoviridae* family but was reclassified by The International Committee on Taxonomy of Viruses (ICTV) in 2016 as a separate family with two genera, *Metapneumovirus* (which includes human metapneumovirus, hMPV) and *Orthopneumovirus* (which includes respiratory syncytial virus, RSV) (Rima et al., 2017).

RSV causes acute respiratory tract illness (ARTI) in individuals of all age groups (Nam and Ison, 2019; Li et al., 2022). The majority of children acquire the infection by the age of two, and recurrent infections are frequently observed (Boivin et al., 2002; Nduaguba et al., 2023). The hMPV virus is capable of causing respiratory tract infections in individuals across all ages, with symptomatic disease being most commonly observed among young children and older adults (Boivin et al., 2002). Many children who are infected by RSV or hMPV are typically less than one year old (Canducci et al., 2008; Wang et al., 2020).

RSV is responsible for seasonal outbreaks of respiratory tract illness worldwide, typically occurring during the winter months (Obando-Pacheco et al., 2018). hMPV exhibits a seasonal pattern, with peak activity in late winter and early spring in temperate regions, and late spring and summer in semitropical areas (Haynes et al., 2016). The multifaceted nonpharmaceutical interventions implemented during the COVID-19 pandemic not only reduced the transmission of SARS-CoV-2 but also impacted the prevalence of other viruses. Studies have shown that the COVID-19 pandemic influenced the prevalence of RSV and hMPV (Yeoh et al., 2021; Kivit et al., 2022).

RSV can lead to severe lower respiratory tract disease, including bronchiolitis, bronchospasm, pneumonia, and acute respiratory failure in pediatric patients (Nair et al., 2010). Lower respiratory tract disease usually occurs with primary infection and may manifest in over 50% of secondary infections (Glezen et al., 1986). hMPV mainly affects children who are asymptomatic or have an upper or lower respiratory tract infection. The most common reasons for hospitalization in hMPV-infected children are acute bronchiolitis and pneumonia (Boivin et al., 2002; Wang et al., 2020). The severity of the disease may be heightened in cases of concurrent infection with RSV. Among children under two years old infected with RSV, co-infection with hMPV has been linked to severe RSV bronchiolitis and the necessity for admission to the intensive care unit (Semple et al., 2004).

The COVID-19 outbreak initially emerged in Wuhan City, China in early 2020 and subsequently disseminated to numerous countries worldwide. Commencing from February 2020, Guangdong Province implemented a series of nationwide measures aimed at curtailing the transmission of COVID-19. These encompassed home quarantine, compulsory mask usage in public settings, alongside other stringent lockdown restrictions. These preventive measures have been enforced for over two years until their cessation in December 2022. The current knowledge regarding the epidemiological characteristics of *pneumoviridae* family viruses following the COVID-19 pandemic is limited compared to pre-pandemic data, and there have been few comprehensive studies assessing the clinical features of severe RSV and hMPV viral pneumonia, as well as their co-infection patterns in children. In this study, we conducted an investigated into the prevalence of RSV and hMPV among children with ARTI in Southern China over a span of twelve years, encompassing both pre- and post-control measures implemented during the COVID-19 pandemic. Understanding the co-infection patterns of common respiratory pathogens in ARTI patients, as well as identifying the clinical characteristics of severe cases, can provide valuable guidance to clinicians for implementing appropriate treatment and prevention strategies.

## Methods

### Patients and specimens

The study was conducted retrospectively on 155,165 hospitalized children 0–17 years of age in Guangzhou Women and Children`s Medical Center (GWCMC) diagnosed with acute respiratory tract illness (ARTI) from 2012 to 2023. The ARTI encompasses upper respiratory tract infections, which primarily affect the nasal passages and pharynx, as well as lower respiratory tract infections involving the trachea, bronchi, and lungs. The presence of cold or cough with or without fever may be accompanied by rapid breathing or difficulty in breathing (Pocket Book of Hospital Care for Children: Guidelines for the Management of Common Childhood Illnesses, 2013). A severe infection case was defined as any case of respiratory tract infection that requires admission to the intensive care unit (ICU) due to its severity. The decision to transfer a patient to the ICU in clinical practice is based on the severity of their condition and their need for advanced life support, as determined by the attending physicians. The nasopharyngeal swab or sputum was taken within 24 hours of admission, and stored in a virus preservation solution and subsequently delivered to the laboratory for detecting within 24 hours.

### Real-time PCR for RSV, hMPV, and other respiratory pathogens viral

The nucleic acids were extracted directly from respiratory specimens using commercially available kits or automated equipment for nucleic acid extraction (Shenzhen Huiyan Kechuang Biotechnology Co., China), according to the manufacturer’s instructions. The viral nucleic acids of RSV, hMPV, and other respiratory pathogens including influenza A virus (IAV), influenza B virus (IBV), parainfluenza virus (PIV), enterovirus (EV), human adenovirus (HAdV), human bocavirus (HBoV), rhinovirus (RHV), *Mycoplasma pneumoniae* (MP), and *Chlamydia pneumoniae* (CP) were detected using quantitative real-time polymerase chain reaction analysis in accordance the corresponding manufacture’s protocols (Guangzhou HuYanSuo Medical Technology Co., China).

### Statistical analysis

SPSS 26.0 software and GraphPad prism 9 were used for data analysis. Categorical data were tested using the Chi-square test and Fisher’s exact test. The Chi-square trend test is employed to conduct trend analysis on incidence rates. Wilcoxon’s test and independent-samples *t*-test were used to analyze continuous variables. Logistic regression analysis was used for predicting risk factors for those infected RSV and hMPV. For all tests, a *p* value < 0.05 was considered as significant.

## Results

### Demographic characteristics

A total of 155,165 pediatric hospitalization patients with ARTI were included in the study from 2012 to 2023. Nasopharyngeal swabs or sputum samples were collected from all these patients, among whom 14,564 tested positive for RSV and 2,524 were found to be positive for hMPV. The median age of ARTI patients was 21 months (interquartile range [IQR], 10–42 months), and the male-to-female gender ratio was 1.76:1. Among RSV- and hMPV-positive patients, the median ages were 8 months (IQR, 3–19 months) and 27 months (IQR, 10–43 months), respectively. The male to female ratios were observed as 1.95:1 in RSV-positive patients and 1.61:1 in hMPV-positive patients.

### Yearly distribution

**Figure 1:**
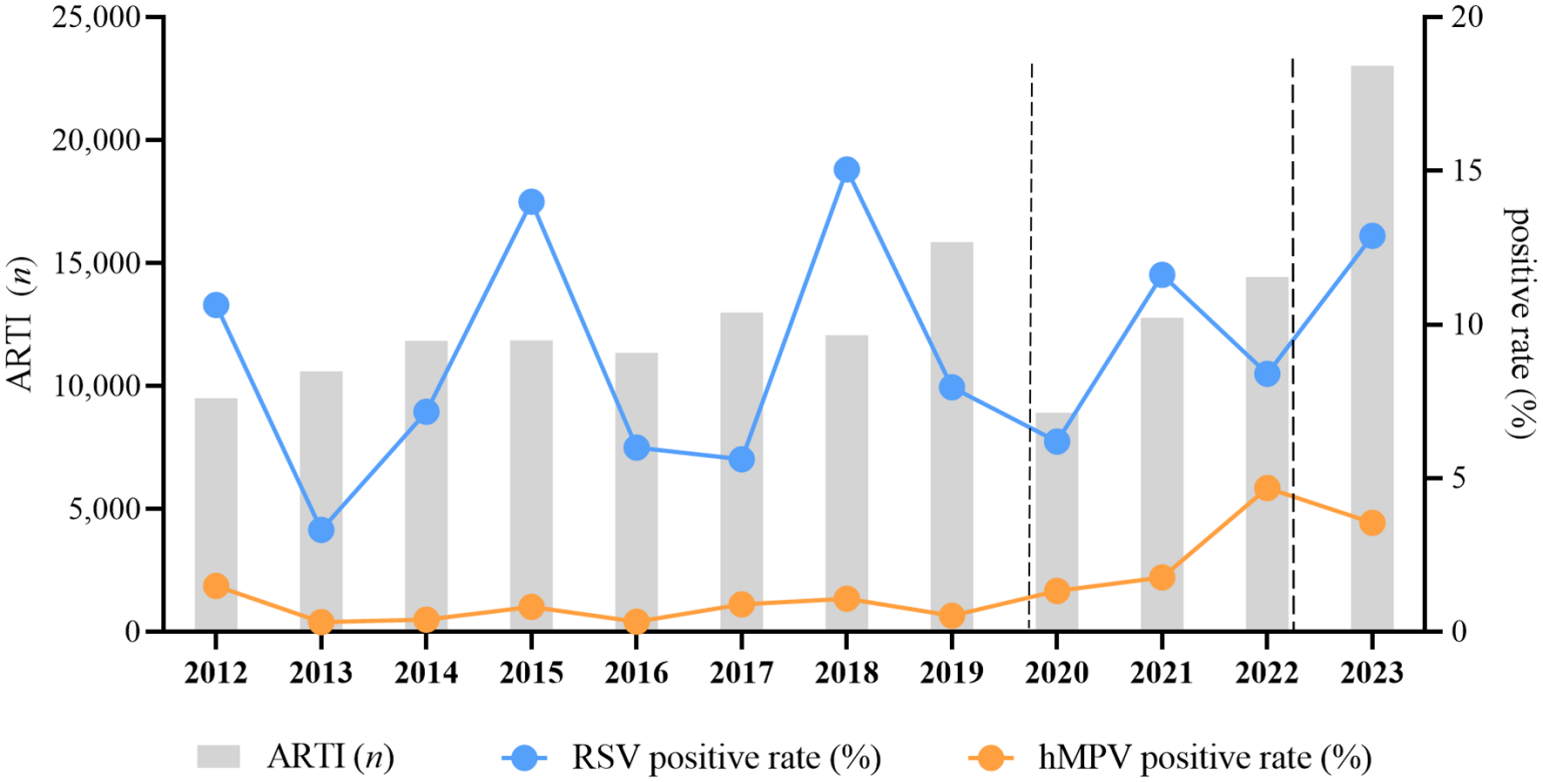
Pediatric hospitalizations with ARTI and the detection rate for RSV and hMPV at GWCMC, 2012–2023. The X-axis represents the timeline of the study. The left Y-axis represents the number of total ARTI samples received and the right Y-axis represents the positive rate of RSV and hMPV.The time period indicated between the two dashed lines represents the duration during which the COVID-19 prevention and control measures are being implemented. The chi-square trend test is utilized for conducting trend analysis on incidence rates, and the results of the test indicate a significant linear trend in RSV (*X^2^* = 272.562, *p*<0.0001) and hMPV (*X^2^* = 1098.864, *p* < 0.0001) incidence across different years. ARTI, acute respiratory tract illness; RSV, respiratory syncytial virus; hMPV, human metapneumovirus; *no*., number; GWCMC, Guangzhou Women and Children’s Medical Center.

The number of ARTI cases ranged from 9,504 to 15,842 before the COVID-19 pandemic. It reached its lowest point at 8,914 in 2020 and then increased to 23,019 in 2023. Cumulatively, a total of 14,564 (9.39%) specimens tested positive for RSV in this study. The rates of RSV positivity ranged from 3.32% in 2013 to 15.04% in 2018. Overall, hMPV was detected in 2,524 specimens (1.63%), with rates ranging from 0.32% in 2013 to 4.68% in 2022 (**Figure 1**). The prevalence of RSV was observed during the winter and spring seasons or during the summer and autumn seasons of the same year, whereas hMPV predominated in the spring season. Before 2017, the prevalence of RSV peaked during the months of January to April; however, post-2017, the majority of peaks shifted to September. The incidence of hMPV infections typically peaks from November to May of the subsequent year, with a limited number of cases occurring between June and September (**Figure 1**).

The trends in the annual total number of specimens tested and positive rates of RSV (*X* ^2^ = 1098.864, *p* < 0.0001) and hMPV (*X^2^* = 272.562, *p* < 0.0001) are illustrated in **Figure 1**, with consistently higher positivity observed for RSV compared to hMPV. The prevalence rates of RSV, occurring every three years, were recorded as 10.63%, 13.99%, and 15.04% in the years 2012, 2015, and 2018 respectively. Similarly, the temporal pattern of hMPV epidemic peak closely resembles that of RSV.

The incidence of ARTI in children exhibited a significant decrease from 2020 to 2022. However, the prevalence of RSV and hMPV did not show a significant decline during the epidemic period characterized by the emergence of SARS-CoV-2 and the implementation of associated non-pharmaceutical interventions and community mitigation measures. In fact, there was a minor surge in RSV epidemic in 2021, while a noticeable upward trend was observed for hMPV epidemic.

### Monthly and seasonal distribution

**Figure 2:**
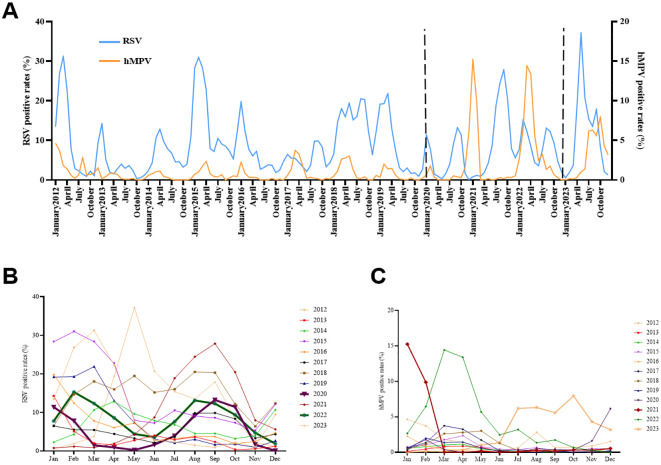
Monthly and seasonal distribution of RSV and hMPV infection in Pediatric hospitalizations with ARTI at GWCMC, 2012–2023. **(A)** Monthly distribution of the RSV- and hMPV-positive rate. The X-axis represents the timeline of the study. The left Y-axis represents the RSV positive rate received and the right Y-axis represents the positive rate of hMPV. The time period indicated between the two dashed lines represents the duration during which the COVID-19 prevention and control measures are being implemented. **(B)** Seasonal distribution of RSV and **(C)** hMPV infection in Pediatric hospitalizations with ARTI. ARTI, acute respiratory tract illness; RSV, respiratory syncytial virus; hMPV, human metapneumovirus; *no*., number, GWCMC, Guangzhou Women and Children’s Medical Center.

The prevalence of RSV and hMPV was assessed monthly over a twelve-year period, from January 2012 to December 2023. The occurrence of RSV infections in children with ARTI is generally observed throughout the year (**Figure 2A**). However, compared to hMPV, the RSV epidemic typically demonstrates an earlier onset and a more prolonged duration. The seasonal peak of hMPV exhibited a slight temporal delay or overlap with that of RSV during the study period. In the years 2017–2019 and 2021, there was a distinct contrast as the peak of hMPV infection preceded that of RSV infection. The prevalence of RSV typically exhibits a single annual peak; however, in 2020, we observed dual peaks occurring in January and September, as well as February and August of 2022 (**Figure 2B**). Similarly, hMPV traditionally displays a solitary peak during the spring season; nevertheless, following the COVID-19 pandemic, it has demonstrated peaks throughout the spring, fall, and winter seasons (**Figure 2C**). Noteworthy, the peak infection rates of RSV and hMPV occurred during and after the implementation of COVID-19 epidemic prevention and control measures. Specifically, the highest RSV infection rate (37.16%) was recorded in May 2023, while January 2021 witnessed the peak hMPV infection rate (15.24%) (**Figure 2A**).

### Gender and age distribution

**Figure 3:**
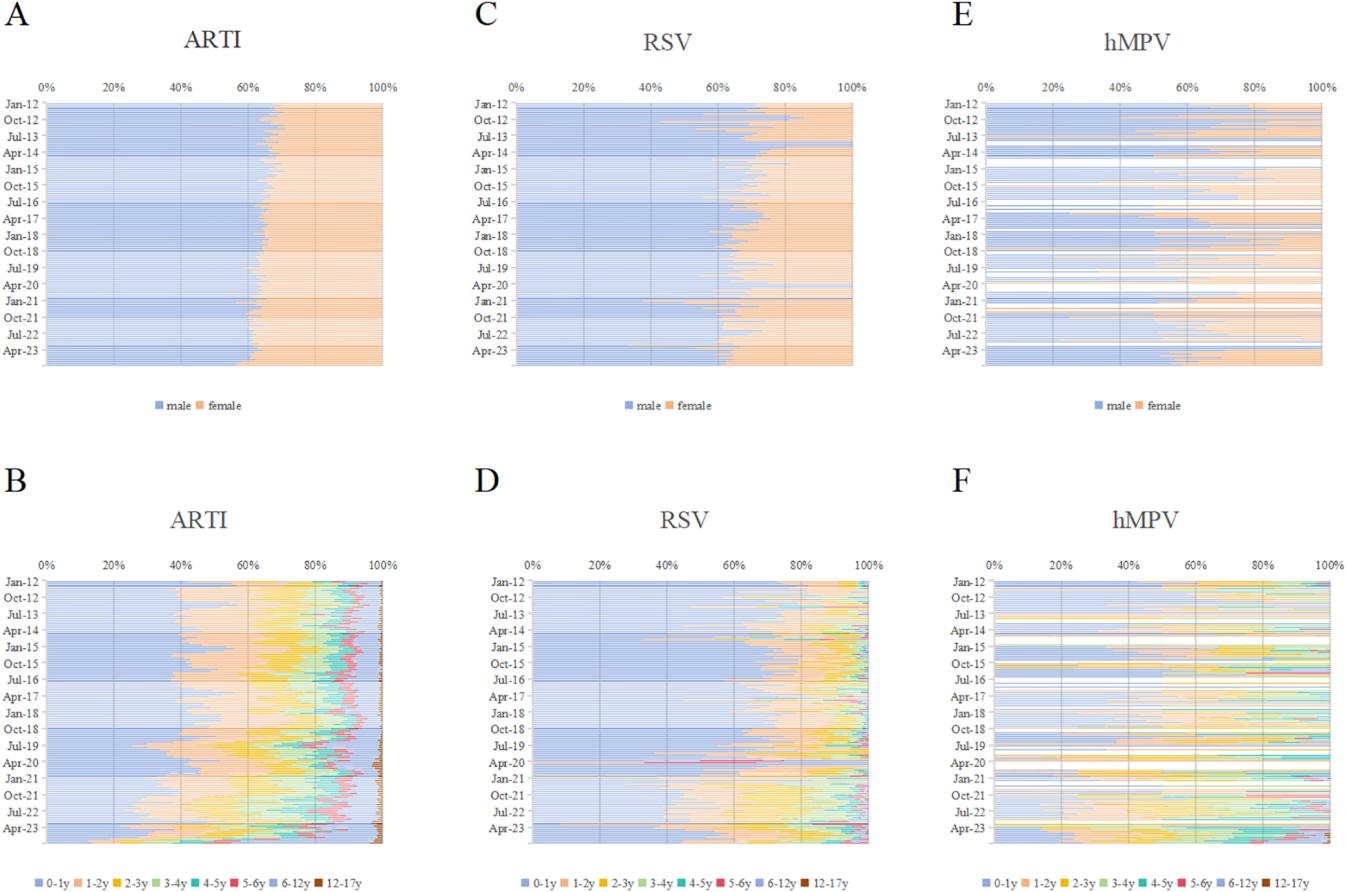
Monthly distribution of RSV and hMPV infections by gender and age in Pediatric hospitalizations with ARTI at GWCMC, 2012–2023. Monthly distribution of ARTI by **(A)** gender and **(B)** age group. Monthly distribution of RSV infections by **(C)** gender and **(D)** age group. Monthly distribution of hMPV infections by **(E)** gender and **(F)** age group. The X-axis represents the percentage of individuals within specific gender or age categories, while the Y-axis denotes each month spanning from 2012 to 2023. ARTI, acute respiratory tract illness; RSV, respiratory syncytial virus; hMPV, human metapneumovirus; *no*., number; GWCMC, Guangzhou Women and Children’s Medical Center; y, year(s).

Around two-third of ARTI, RSV-, and hMPV- positive cases were male (**Figures 3A, C, E**). The gender distribution of ARTI, RSV, and hMPV infections did not exhibit significant differences.

The proportion of ARTI cases in infants aged 0–1 year was 37.85% (58,730/155,165) (**Figure 3B**). Meanwhile, the prevalence of RSV- and hMPV-positive cases in this age group accounted for 60.86% and 28.61% (**Figures 3D, F**). The frequency of RSV cases was significantly higher in infants (0–1-year-old) compared to hMPV, indicating a contrasting pattern. (*p* < 0.0001). The age distribution of RSV and hMPV infections was significantly different (*p* < 0.001). The trends in monthly gender and age ratios of ARTI, RSV, and hMPV positive patients are illustrated in **Figure 3**. Following the COVID-19 epidemic, an increasing proportion of female patients and individuals aged one year and older has been observed.

### Co-infection of other common respiratory pathogens and co-infection in severe cases

**Figure 4:**
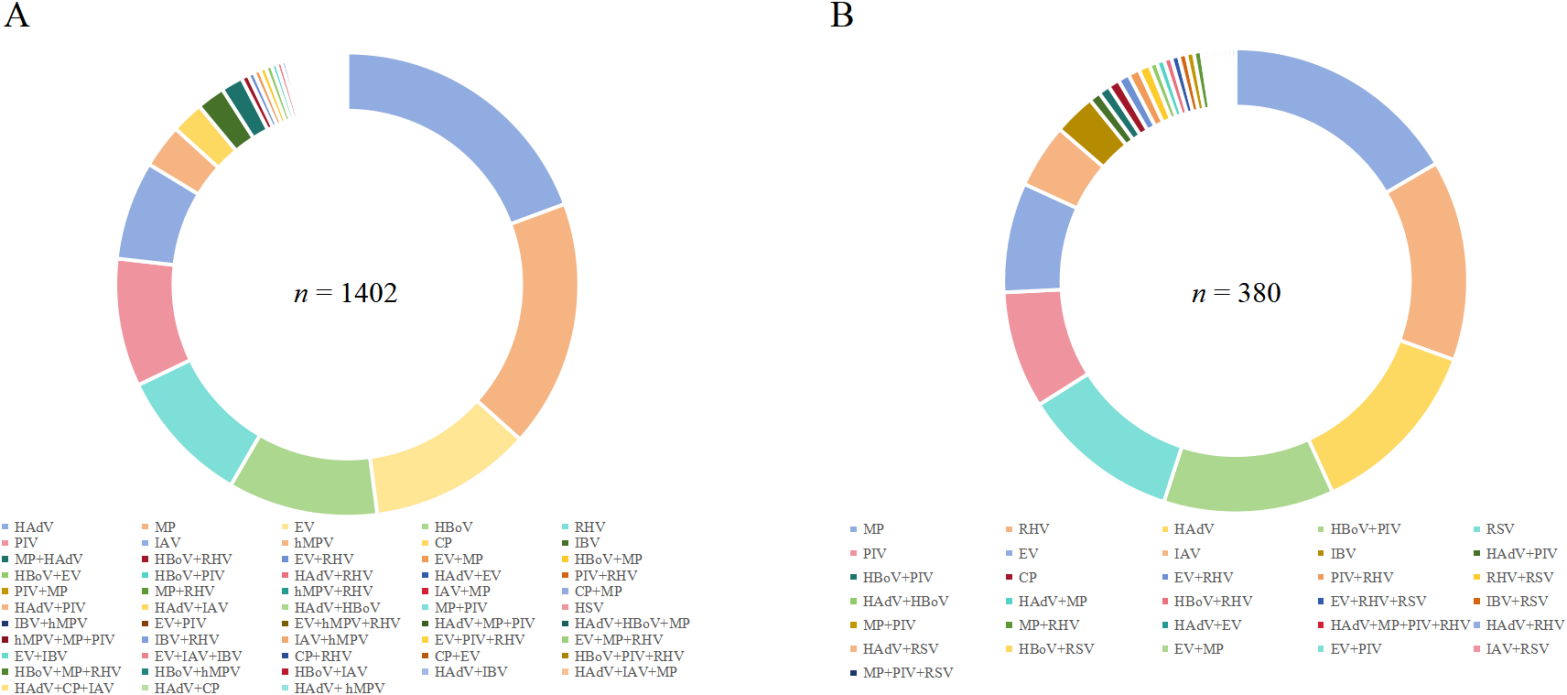
Percentage of co-infection of **(A)** RSV and **(B)** hMPV with other respiratory pathogens detected in Pediatric hospitalizations with ARTI at GWCMC, 2012–2023. ARTI, acute respiratory tract illness; RSV, respiratory syncytial virus; hMPV, human metapneumovirus; *n*, number; GWCMC, Guangzhou Women and Children’s Medical Center; AdV, human adenovirus; RHV, rhinovirus; RSV, respiratory syncytial virus; MP, *Mycoplasmapneumoniae*; HBOV, human bocavirus; PIV, parainfluenza virus type 1 and 3; IFVA, influenza virus types A; IFVB, influenza virus types B; EV, enterovirus; and CP, *Chlamydia pneumonia*.

The detection rates of viral or atypical pathogen co-infections in cases positive for RSV and hMPV were 9.63% (1,402/14,564) and 15.06% (380/2,524), respectively (**Figure 4**).

Among 1,402 patients who tested positive for RSV, the pathogens most frequently detected as co-infections were HAdV (19.04%, 267/1,402), MP (16.98%, 238/1,402), and EV (11.13%, 156/1,402). Notably, 8.92% (125/1,402) of patients had concurrent infections with two or more pathogens (**Figure 4A**). Additionally, ICU treatment was required for 6.86% (69 cases) of RSV-positive patients with co-infections (**Table 1**).

**Table 1:** Univariate analysis of baseline characteristics of hospitalized children infected with RSV and hMPV.

Categories	RSV, *no*. (%)					hMPV, *no*. (%)				
	Non-ICU	ICU	OR	95%CI	*p* value	Non-ICU	ICU	OR	95%CI	*p* value
**Hospital admission for RSV or hMPV infected**	13558 (93.09)	1006 (6.91)				2465 (97.66)	59 (2.34)			
Gender*			0.918	0.803–1.050	0.211			1.129	0.658–1.935	0.659
Male	8982 (66.25)	647 (64.31)				1518 (61.58)	38 (64.41)			
Female	4576 (33.75)	359 (35.69)				947 (38.42)	21 (35.59)			
**Age group**					<0.0001^#^					0.0078^#^
0–1 y	7966 (58.75)	898 (89.26)	5.837	4.767–7.147	<0.0001**	699 (28.36)	23 (38.98)	1.614	0.950–2.744	0.074**
Before the COV-19 epidemic	5424 (68.09)	491 (54.67)				323 (46.21)	13 (56.52)			
During the COV-19 epidemic	1337 (16.78)	151 (16.82)			<0.0001^#^	204 (29.18)	4 (17.39)			0.463^#^
After the COV-19 epidemic	1205 (15.13)	256 (28.51)				172 (24.61)	6 (26.09)			
1–2 y	2493 (18.39)	69 (6.86)				487 (19.76)	11 (18.64)			
2–3 y	1499 (11.06)	14 (1.39)				393 (15.94)	8 (13.56)			
3–4 y	944 (6.96)	12 (1.19)				423 (17.16)	4 (6.78)			
4–5 y	318 (2.35)	4 (0.40)				257 (10.43)	5 (8.47)			
5–6 y	134 (0.99)	3 (0.30)				90 (3.65)	4 (6.78)			
6–12 y	182 (1.34)	5 (0.50)				108 (4.38)	4 (6.78)			
13–16y	22 (0.16)	1 (0.10)				8 (0.32)	0 (-)			
Co-infection*	1333 (9.83)	69 (6.86)	0.675	0.525–0.868	0.0002	369 (14.97)	11 (18.64)	1.302	0.670–2.530	0.459
Underlying medical conditions*	1083 (7.99)	115 (11.43)	1.487	1.213–1.823	<0.0001	70 (2.84)	16 (27.12)	12.731	6.840–23.695	<0.0001
Congenital heart disease	201 (1.48)	38 (3.78)	2.609	1.833–3.712	<0.0001	7 (0.28)	1 (1.69)	6.054	0.733–50.007	0.173
Familial genetic disease	183 (1.35)	19 (1.89)	1.407	0.873–2.267	0.161	18 (0.73)	1 (1.69)	2.344	0.308–17.854	0.363
Digestive diseases	126 (0.93)	6 (0.60)	0.640	0.281 –1.454	0.390	5 (0.20)	0	–	–	> 0.9
Hematologic and neoplastic diseases	124 (0.91)	10 (0.99)	1.088	0.569–2.078	0.864	3 (0.12)	1 (1.69)	14.149	1.450–138.072	0.090
After surgery	117 (0.86)	15 (1.49)	1.739	1.012–2.988	0.055	14 (0.57)	1(1.69)	3.018	0.390–23.340	0.299
Protein-energy malnutrition	106 (0.78)	10 (0.99)	1.274	0.664–2.444	0.582	11 (0.45)	4(6.78)	16.225	5.010–52.547	< 0.0001
Central nervous system disease	104 (0.77)	8 (0.80)	1.037	0.504–2.135	0.9	3 (0.12)	7 (11.86)	110.474	27.790–439.173	< 0.0001
Urogenital diseases	94 (0.69)	2 (0.20)	0.285	0.070–1.159	0.066	9 (0.37)	0	–	–	> 0.9
Endocrine system diseases	28 (0.21)	7 (0.70)	3.386	1.475–7.771	0.009	0	1 (1.69)	–	–	0.023

RSV, respiratory syncytial virus; hMPV, human metapneumovirus; ICU, intense care unit; y, year(s); *no.*, number.

Chi-square test or Fisher’s exact test showed that the proportion of RSV- or hMPV-positive children treated in the ICU was significantly higher than that treated in the inpatient (non-ICU).

*Chi-square test or Fisher’s exact test.

^#^Wilcoxon test.

**The Fisher’s exact test of the disparity in ICU admission rates among children aged 0–1 years following infection with RSV or hMPV, as compared to those aged 1 year and above.

Among hMPV-positive patients, the three predominant pathogens causing co-infections were MP (16.58%, 63/380), RHV (13.95%, 53/380), and HAdV (12.63%, 48/380). A total of forty-one cases (10.79%) exhibited concurrent infection with two or more pathogens (**Figure 4B**). The proportion of hMPV-positive patients requiring ICU admission who had co-infections was found to be18.64% (11/59) as shown in **Table 1**.

### Demographic and clinical characterization of the severity in cases positive for RSV and hMPV

Demographic data (gender and age), the presence of pathogens co-infections, and the types of comorbidities were categorized based on the severity of RSV and hMPV positive patients (**Table 1**).

The admission rates for RSV- and hMPV positive cases in ARTI patients were 6.48‰ (1,006/155,165) and 0.38‰ (59/155,165), respectively, when admitted to the ICU.

The comparison of RSV and hMPV results between the non-ICU and ICU subgroups did not reveal any significantly differences in terms of gender. (*p* = 0.211 and 0.659, respectively, **Table 1**).

The age distribution of RSV and HMPV infections was significantly difference (*p* < 0.0001, **Table 1**); hMPV patients’ median age was higher; a decreasing trend of epidemic infection was observed with increasing age among children infected with RSV and hMPV, both in non-ICU and ICU settings. The frequency of ICU cases among RSV- positive infants (0–1 year) was significantly higher compared to non-ICU cases (*p* < 0.0001). There was no statistically significant difference in the proportion of hMPV-positive children under three years old compared to those over three years old, for both ICU and non-ICU cases (*p* = 0.2753) (**Table 1**). We categorized infants infected with RSV and hMPV into three groups: before, during, and after the implementation of the COVID-19 prevention strategy. Infants who encountered their initial RSV infection after the relaxation of epidemic control measures displayed more severe symptoms compared to those before the measures were lifted (*p* < 0.0001). However, there was no significant disparity in symptoms among infants experiencing their first hMPV infection at different epidemic control measures stages (*p* = 0.463) (**Table 1**).

Other respiratory pathogens co-infection was more frequent in the group with severe hMPV infection (*n* = 11) (18.64% vs 14.97%, *p* = 0.459), compared to those who did not have it. On the contrary, the rate of co-infection in patients with severe RSV infection was significantly lower at 6.86% compared to non-severe infection patients at 9.83%, and the comparative results showed high statistical significance (*p* = 0.0002) (**Table 1**).

In severe RSV patients, the prevalence of comorbidity was observed to be 11.43% (115/1,006), whereas in severe hMPV patients, the prevalence of comorbidity reached as high as 27.12% (16/59), which exhibited a significantly higher rate compared to that in mild patients (*p* < 0.0001 for both) (**Table 1**).

The proportion of children with congenital heart disease, surgery, and endocrine system diseases who tested positive for RSV and were admitted to the ICU was higher compared to RSV-infected children in non-ICU settings (*p* < 0.0001, *p* = 0.055, and *p* = 0.009, respectively) (**Table 1**). However, a significant percentage of critically ill children with hMPV infection admitted to the ICU had protein-energy malnutrition and central nervous systen diseases (*p* <0.0001 for both) (**Table 1**).

### Correlation of the severity in cases positive for RSV and hMPV

Logistic regression analysis was utilized to examine the association between demographic and clinical characteristics and the severity of RSV and hMPV positivity. The incidence of cases attributed to hMPV in the ICU is relatively low. Therefore, the independent variables included in the final logistic regression analysis were age, presence or absence of co-infection, and presence or absence of comorbidities. The analysis findings suggest that infant (OR = 4.767, 95%CI: [3.888–5.846], *p* < 0.0001), presence of co-infection (OR = 0.540, 95%CI: [0.404–0.722], *p* < 0.0001), and existence of comorbidities (OR = 1.582, 95%CI: [1.285–1.949], *p* < 0.0001) was the risk ratio for the severity of RSV infection. Additionally, the severity of hMPV infection was significant correlated with the patients under one year of age (OR = 0.322, 95%CI: [0.180–0.575], *p* < 0.0001) and patients presented with comorbidities (OR = 8.809, 95%CI: [4.493–17.272], *p* < 0.0001) (**Table 2**). The analysis of the severity of RSV infection with comorbidities also included six comorbidities that may have exhibited variations in the univariate analysis, serving as independent variables. The presence of congenital heart disease (OR = 2.789, 95%CI: [1.626–4.783], *p* < 0.0001) or endocrine system diseases (OR = 3.687, 95%CI: [1.461–9.306], *p* = 0.006) in patients hospitalized with RSV infection constitutes risk factors for the severity of the infection (**Table 2**). The incidence of cases attributed to hMPV in the ICU is relatively low; therefore, no effective logistic regression analysis was established of comorbidities and the severity of hMPV infection.

**Table 2:** Logistic regression analysis of correlation between severity of RSV, hMPV and baseline characteristics factors.

Dependent variable	Independent variable	OR	95%CI for OR	*p* value
Admit in ICU for RSV infection	Age (< 1 y)	4.767	3.888–5.846	< 0.0001
	Co-infection	0.540	0.404–0.722	< 0.0001
	Underlying medical conditions	1.582	1.285–1.949	< 0.0001
Admit in ICU for hMPV infection	Age (< 1 y)	0.322	0.180–0.575	< 0.0001
	Co-infection	0.601	0.297–1.216	0.157
	Underlying medical conditions	8.809	4.493–17.272	< 0.0001
Admit in ICU for RSV infection with underlying medical conditions	Congenital heart disease	2.789	1.626–4.783	< 0.0001
	Familial genetic disease	1.531	0.817–2.869	0.183
	After surgery	1.891	0.960–3.726	0.066
	Premature birth/malnutrition	1.392	0.645–3.002	0.400
	Urogenital diseases	0.314	0.073–1.352	0.120
	Endocrine system diseases	3.687	1.461–9.306	0.006

CI, confidence interval; OR, odds ratio; ICU, intense care unit; RSV, respiratory syncytial virus; hMPV, human metapneumovirus; y, year. Logistic regression analysis was performed for the severity of RSV and hMPV positive as the dependent variable, with age, presence of co-infection, and existence of underlying medical conditions considered as independent variables.

## Discussion

The present study conducted a retrospective analysis of the ARTI seasons spanning from 2012 to 2023, encompassing both pre- and post-pandemic periods of SARS-CoV-2, with a focus on laboratory and clinical data pertaining to cases of RSV and hMPV.

The occurrence of four epidemic seasons of RSV was observed between 2012 and 2023, with each season recurring at three-year intervals (**Figure 1**), as previously reported (Hamid et al., 2023; Li et al., 2023). The Chi-square trend test results indicate a statistically significant linear trend in the incidence of RSV across different years, suggesting an anticipated surge in RSV infections among pediatric populations in the year 2024. Similar to numerous other respiratory viruses (Chow et al., 2022), RSV exhibited lower-than-anticipated levels in 2021 subsequent to the emergence of SARS-CoV-2 and the ensuing pandemic caused by associated nonpharmaceutical interventions and community mitigation. The aspect that intrigued us was the remarkable finding that despite the implementation of COVID-19 prevention and control policies, there was a documented rise in the prevalence of hMPV among pediatric patients (**Figure 1**). The observed increase may be attributed to the continuous cycles of mild or asymptomatic reinfection of hMPV within families and communities, as well as the prolonged shedding of infectious virus from hMPV-infected recovering patients. Therefore, we suggest that immunocompromised patients should avoid close contact with individuals infected with hMPV or those who have recently recovered from the infection. For hospitalized patients diagnosed with hMPV, strict contact precautions should be implemented, and they should be isolated in a dedicated room or cohorted with other patients also affected by hMPV.

The seasonal outbreaks of RSV and hMPV are commonly observed worldwide (Wang et al., 2020; Li et al., 2022; Perez et al., 2022). In the Northern Hemisphere, outbreaks of RSV typically occur from October or November to April or May of the subsequent year, with a peak observed in January or February; In the Southern Hemisphere, the winter epidemic of RSV infection transpires between May and September, reaching its zenith in May, June, or July (Staat et al., 2013; Obando-Pacheco et al., 2018). The infection of hMPV also exhibits a seasonal pattern. In Europe and North America, hMPV infection predominantly manifests during the late winter and early spring seasons (McAdam et al., 2004; Haynes et al., 2016). Studies conducted in mainland China have demonstrated that the peak prevalence of hMPV occurs during the late spring and summer periods (Chiu et al., 2022; Cui et al., 2022). In the present study, the annual distribution of RSV and hMPV cases exhibited an equal seasonal pattern (**Figure 2A**). The prevalence peak of RSV typically occurs in the spring and fall, exhibiting a single annual peak; meanwhile, hMPV circulation is higher during the months of January to March, consistent with prior research. However, the outbreak of the COVID-19 pandemic, however, has caused a shift in these patterns of prevalence. Our findings revealed two distinct peaks of RSV infection during the COVID-19 pandemic, occurring in January and September 2020, as well as in February and August 2022 (**Figure 2B**). The incidence of hMPV infection reached its highest point during the winter of 2020 and autumn of 2023 (**Figure 2C**).

The analysis of patients with ARTI demographic data substantiated the prevailing notion that infants are more susceptible to and experience more severe cases of RSV-related infections, with nearly 60% of RSV cases occurring in children under one year old (**Figure 3**). After the relaxation of COVID-19 control measures, infants who have not previously contracted RSV may present with more severe symptoms. The severity of initial infections in infants may be attributed to the implementation of control measures during the pandemic, which effectively curtailed virus transmission and hindered the development of immune memory through mild infections. These findings imply a greater public health burden on infants and young children, as previously documented (Li et al., 2022). The distribution of hMPV-associated illnesses across pediatric age groups was found to be more evenly balanced compared to RSV (**Figure 3**), aligning with the findings of a recent multicenter study conducted in the United States (Nadiger et al., 2023). The severity of infants’ first hMPV infection did not exhibit any discernible differences across various stages of COVID-19 control measures. However, it is important to note that the limited availability of data on hMPV infection in infants may have influenced this outcome. Although the majority of individuals acquire RSV infection by the age of 2 years and hMPV infection by the age of 5 (Glezen et al., 1986; van den Hoogen et al., 2001), we have observed an increase in the proportion of older children and female patients among hospitalized individuals with ARTI, specifically those caused by RSV and hMPV, subsequent to the COVID-19 outbreak (**Figures 3B, D, F**). The speculation arises that both RSV and hMPV-associated respiratory diseases may potentially cause outbreaks among the pediatric population in the foreseeable future. Therefore, it is recommended to expand preventive and clinical management measures targeting RSV and hMPV to encompass children of all age groups.

The co-infection rates of RSV and hMPV with MP were both significantly elevated (**Figure 4**), which is consistent with the findings of previous studies (Jain et al., 2015). This observation may be attributed to the persistent rise in the incidence of Macrolide-resistant *M. pneumoniae* (Kim et al., 2022; Meyer Sauteur et al., 2022), resulting in delays in effective antibiotic treatment and prolonged hospitalization stays. The shedding of HRV in infants has been observed to persist for over 2 weeks following infection (Jartti et al., 2008), potentially contributing to the elevated rates of RHV co-infection with RSV or hMPV.

In this study, it is noteworthy that 6.91% and 2.34% of ARTI patients infected with RSV and hMPV of all ages required admission to intensive care (**Table 1**). Although infants under one year of age are widely acknowledged to be at a heightened risk for RSV infection and serve as a significant source of severe consequences following infection (Hall et al., 2009), it has not been previously undertaken to systematically analyze the disease burden and risk factors associated with RSV and hMPV infections in pediatric populations. The results of our study suggest that infants who has respiratory pathogens coinfected with RSV or underlying medical conditions are at a heightened risk for developing severe disease associated with RSV (**Table 2**).

The latest research findings indicate that underlying medical conditions play a pivotal role in determining the severity of outcomes associated with RSV infection (Wang et al., 2024). The results of logistic regression analyses exploring the prevalence of coexisting conditions highlight that patients with RSV infection and underlying long-term effects (such as congenital heart disease and endocrine disorders) are at a higher risk of critical illness compared to those who have experienced mild RSV infection (**Table 2**).

The incidence of hMPV infection in the ICU is relatively low. Therefore, a valid logistic regression analysis to assess the relationship between comorbidities and the severity of hMPV infection could not be established. However, univariate analysis revealed that infants under one year old or patients with underlying diseases may be at higher risk for developing critical illness associated with hMPV infection (**Table 1**). The discovery holds significant implications for clinicians seeking to enhance the management of severe RSV and hMPV infections in children.

There are some certain limitations of this study that should be acknowledged. 1) The scope of this study was limited to hospitalized children with acute respiratory infections in southern China, excluding children with acute respiratory infections in northern China and outpatients. Therefore, the findings may not be generalizable to the entire Chinese population. 2) The rarity of severe cases of hMPV hindered a multifactorial analysis of the underlying disease in patients with severe hMPV. 3) Due to insufficient data, we were unable to assess the effects of bacterial or fungal co-infection, critical condition patient outcomes, and household economic background on the risk of RSV and hMPV-associated ARTI. Therefore, none of the associations identified in this study can be interpreted as causal relationships. The aforementioned limitations will serve as a valuable foundation for future prospective multicenter studies aimed at evaluating the impact of acute respiratory infection risk in comparison to various models within a more diverse and applicable population.

In conclusion, our findings suggest that the prevalence of RSV and human metapneumovirus hMPV following the lifting of the COVID-19 pandemic restrictions may exhibit variations in comparison to previous seasonal patterns, with potential deviations in terms of sex and age distribution specificity. These changes could potentially lead to widespread severe outbreaks among children, particularly those with underlying medical conditions who may experience more severe symptoms. Consequently, our findings offer a reliable foundation for future epidemic prevention and treatment strategies.

## Data Availability

The raw data supporting the conclusions of this article will be made available by the authors, without undue reservation.
